# Marjolin's ulcers: theories, prognostic factors and their peculiarities in spina bifida patients

**DOI:** 10.1186/1477-7819-8-108

**Published:** 2010-12-05

**Authors:** Peter M Nthumba

**Affiliations:** 1 Department of Surgery, AIC Kijabe Hospital, Kijabe, Kenya, Africa

## Abstract

**Background:**

Due to improved care, more and more children born with spina bifida in rural Kenya are surviving into adulthood. This improved survival has led to significant challenges in their lifestyles, especially the need to ensure pressure ulcer prevention and treatment. Malignant degeneration of pressure ulcers in spina bifida patients is very rare. The author describes the clinical presentation of two pressure ulcer carcinomas that are at variance from classical descriptions.

**Materials and methods:**

An internet/Medline/PubMed search of English literature for theories on Marjolin's ulcer evolution and prognostic features of Marjolin's ulcers was performed.

A chart review of two young adults with spina bifida who had presented to the author's hospital between 2004 and August 2010 with chronic pressure ulcers found to be Marjolin's ulcers on histo-pathological examination was performed, and the clinical features are reported.

**Results:**

The two ulcers appeared clinically benign: one was a deep ulcer, while the other was shallow; both had normal, benign-appearing edges, and a foul smelling discharge. The two ulcers were surrounded by induration and multiple communicating sinuses, with no evidence of chronic osteomyelitis. The internet search revealed a total of nine theories on Marjolin's ulcer development, as well as seven clinical and four histological prognostic features.

**Discussion:**

The multifactorial theory, a coalescence of a number of proposed theories, best explains the evolution of Marjolin's ulcers. Poor prognostic features include pressure ulcer carcinomas, lesions and location in the lower limbs/trunks, all present in the two patients making their prognosis dim: this is despite the surgical margins being clear of tumor. Benign appearance, induration and presence of multiple communicating sinuses are features that have not been previously described as presenting features of pressure ulcers carcinomas.

**Conclusion:**

There is need for spina bifida patients and their guardians/caretakers to receive a close follow-up throughout life; health education focused on pressure ulcer prevention as well as early treatment of pressure ulcers when they occur, will avert the development of Marjolin's ulcers, and save lives.

## Background

The population of children with spina bifida surviving into adulthood in rural Kenya is growing because of improved health education, care as well as an increasingly supportive environment [1]. Improved survival and integration into such social structures as schooling, work, marriage and child-bearing places significant demands on this population: the need for a lifestyle that is protective/preventive against the development of such life-threatening complications as renal failure and pressure ulcers, amongst others. Prevention requires active bladder and bowel care, as well as regular shifting of position to avoid prolonged pressure leading to the development of pressure ulcers. Failure to adhere to this 'protective lifestyle' almost invariably leads to the development of pressure ulcers; these ulcers may heal with appropriate care. Others may suffer either frequent ulcer relapses or chronic non-healing ulcers that may degenerate into Marjolin's ulcers. A number of hypotheses have been proposed to explain malignant degeneration of chronic wounds and scar tissue (Table 1) [2-16].

**Table 1:** Theories on Marjolin's ulcers [2-16]

Theory	Proposed mechanism
Toxin theory	Toxins released from damaged tissues later lead to cellular mutations.

Chronic irritation theory	Chronic irritation with repeated attempts at re-epithelialization contributes to neoplastic initiation.

Traumatic epithelial elements implantation theory	Epithelial elements implanted into the dermis, lead to a foreign body response reaction and a disordered regenerative process.

Co-carcinogen theory	Chemical or trauma such as burn injury acts to 'stir' pre-existing but dormant neoplastic cells into proliferation.

Initiation and promotion theory	A two-step process that converts normal cells into malignant cells. In the initiation phase, normal cells become dormant neoplastic cells that may then be subsequently stimulated into neoplastic cells by a co-carcinogen such as infection, in the promotion phase. This theory overlaps with the co-carcinogen theory.

Immunologic privileged site theory	Burn scarring effectively obliterates lymphatics to injured area, preventing normal immunosurveillance and thus permitting neoplastic growth. These tumors initially grow slowly, but quickly overwhelm the immune system, metastasize and are rapidly fatal, once they break through the scar barrier.

Heredity theory	HLA DR4 is associated with cancer development and p53 gene abnormalities have been demonstrated in patients with Marjolin's ulcers. Further, *Fas *mutations in the apoptosis function region that predispose to malignant degeneration of scars have been demonstrated in burn scar Marjolin's ulcers.

Ultraviolet rays theory	Ultraviolet rays theory - UV rays cause a reduction in Langerhans cell population leading to a reduction in cutaneous immuno-surveillance against developing malignancy and also cause p53 tumor suppressor gene alterations.

Environmental and genetic interaction theory	Attempts to explain the occurrence of 'Acute' Marjolin's ulcers.

Four clinical signs have been proposed as characteristic for malignant pressure ulcer degeneration: the appearance of a mass, new onset of pain, a change in drainage odor and change in volume, character or appearance of drainage [17]. Unfortunately, most spina bifida patients lack sensation, and they and their caretakers may not recognize any significant changes in their ulcers. Health education, with an emphasis on ulcer prevention and care, should be taught to healthcare workers and parent(s)/guardian(s); it is ulcers that develop in childhood that may later degenerate into malignancy [18].

Our understanding of the process of pressure ulcer development amongst spina bifida patients, and their subsequent degeneration into malignant ulcers is limited. The purpose of this study was to collect and review the various theories on Marjolin's ulcers, the different prognostic factors, with a view to applying these to spina bifida patients. This understanding would aid the healthcare worker in developing programs suited to a growing population of spina bifida patients, especially in the low income countries. The author also sought to describe atypical clinical presentation of Marjolin's ulcers in these patients.

## Patients and methods

A chart review of two young adults with spina bifida who had presented to the author's hospital between 2004 and August 2010 with chronic pressure ulcers found to be squamous cell carcinomas on histopathological examination was performed.

An internet/Medline/PubMed search of English literature for pressure ulcer theories as well as on the prognostic features of Marjolin's ulcers was performed. The terms 'pressure ulcer', 'pressure sore', 'decubitus ulcer' independently and with the term 'theory' or 'theories' were used, as were the terms, 'Marjolin's ulcers', 'malignant pressure ulcers', 'prognosis', 'prognostic features', in various combinations.

## Results

**Figure 1:**
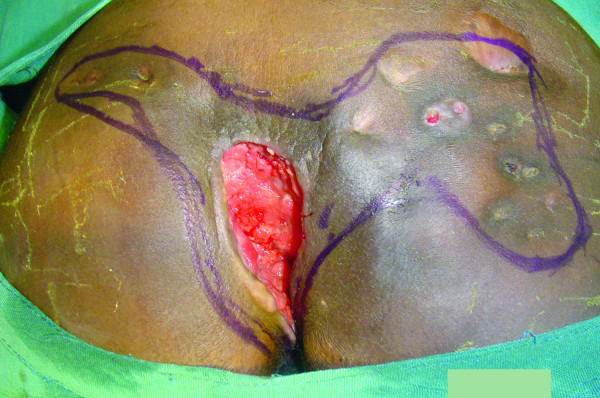
**Marjolin's ulcer with sinuses included within surgical excision margins**. Note deep ulcer and benign appearance of ulcer edges.

**Figure 2:**
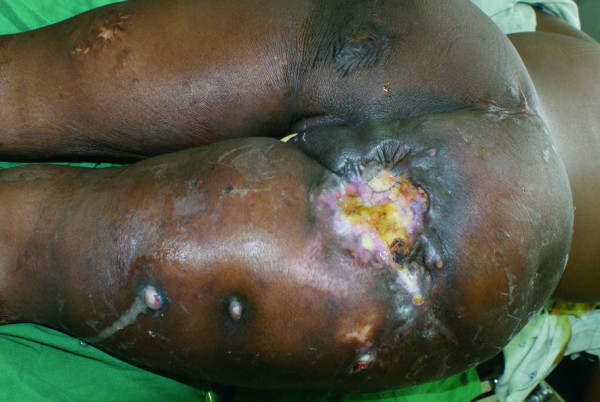
**Marjolin's ulcer with sinuses extending into the thigh and labia majora**. One sinus was found in the anus, and another in the vagina. Note benign appearance of ulcer margins surrounding a flat ulcer.

The two patients, both females, were aged 20 and 26 years. While one of the patients was ambulant with bilateral below-knee prostheses [1], the other was wheelchair-bound. Both had chronic pressure ulcers; one had lasted 16 years, while the second patient had had the ulcer for five years, with a previous history of ulcers from the same site that had recurred a number of times in the past, with none having lasted for more than a year. The ulcer of one patient was deep, while the other was a shallow flat ulcer: both had a foul smelling purulent discharge and multiple sinuses that communicated with the ulcer. The areas with the ulcers and the sinuses were indurated, and on digital pressure exuded discharge both from the ulcer and sinuses. The margins of the ulcers were of normal appearance, (not elevated), and would thus not suggest malignancy to the casual observer (Figure 1 and 2). The excised surgical margins on both patients were clear of tumor. There was no evidence of underlying chronic osteomyelitis.

The internet/Medline/PubMed search on pressure ulcer theories revealed a total of nine different hypotheses (Table 1) [2-16], while a search for prognostic features of Marjolin's ulcers revealed seven clinical and four histological features (Table 2) [19-24].

**Table 2:** Prognostic factors in Marjolin's ulcers [19-24]

		PROGNOSIS	
	**Variable**	**Better**	**Poorer**

Clinical	Latency to malignancy	Less than 5 years	More than 5 years
	
	Tumor location	Head, neck, upper extremeties	Lower limbs, trunk
	
	Tumor source	Post-burn, chronic osteomyelitis	Pressure sore carcinomas
	
	Tumor diameter	Smaller than 2 cm	2 cm or more
	
	Tumor type	Exophytic	Infiltrative
	
	Metastases	None	Present
	
	Tumor recurrence	None	Present

Histological	Degree of differentiation	Well differentiated	Moderately-well and poorly differentiated
	
	Peritumoral T lymphocyte infiltration	Heavy	Scarce or absent
	
	Depth of dermal invasion	Superficial to reticular dermis	Reticular dermis or deeper
	
	Vertical tumor thickness	Less than 4 mm thick	4 mm thick or more

## Discussion

A review of theories on Marjolin's ulcer evolution reveals that no single theory explains their evolution fully. These postulates include the toxin, the chronic irritation, the traumatic epithelial elements implantation, the co-carcinogen and the initiation and promotion theory; these theories include trauma as an integral part of the process of the evolution of Marjolin's ulcers [2-9]. The immunologically privileged site theory, which has a large number of proponents, attempts to explain the poor prognosis of Marjolin's ulcers [10,11]. The hereditary and ultraviolet rays' theories were proposed after genetic changes were found in patients with Marjolin's ulcers [12-15]. The environmental and genetic interaction theory seeks to explain the evolution of acute Marjolin's ulcers [16]. A combination of theories better explains the process: for example, the chronic irritation, the initiation and promotion, the toxin and the co-carcinogen theories when combined together, explain the evolution of pressure ulcer carcinomas, under which spina bifida pressure ulcers fall. The current author proposes the multifactorial theory, a combination of any of the current theories (Table 1) [2-16], as the one that best explains this process. It is to be noted that some of these theories may overlap.

Marjolin's ulcers complicating pressure ulcers in spina bifida patients are rarely reported: there are less than ten reported cases in English literature [1]. Marjolin's ulcers in general, develop in younger patients amongst sub-Saharan patients than those reported from other regions [18]; therefore, patients presenting with pressure ulcers should be investigated during the initial evaluation for this possibility. Additionally, at surgery, all the excised tissue should be submitted for histopathological investigation. Unfortunately, surgical margins clear of malignancy do not necessarily improve the prognosis of pressure ulcer carcinomas [1,18], which have a much poorer prognosis than Marjolin's ulcers arising from other sources [4]. Table 2 highlights prognostic features of Marjolin's ulcers in general - it is notable that a pressure ulcer carcinoma is a poor prognostic indicator. Further, Marjolin's ulcers located on the lower limbs or trunk, those with diameters above two centimeters, and latency of five years or more, all common features in the two spina bifida patients presented here, made their prognosis even poorer, especially in an environment with limited resources and options [1,3,11,19-24].

Marjolin's ulcers are characteristically either grossly flat, indurated, infiltrative shallow ulcers with well-defined, elevated margins, or exophytic proliferative ulcers [1]. The two ulcers in this report had a benign appearance of both the ulcer edges and the bases, and except for a foul smell, none of the other four hallmark signs of pressure ulcer carcinoma [17] were found. The other common features in these two ulcers were: induration and multiple sinuses communicating with the ulcers, two signs that have not been previously noted in pressure ulcer carcinomas. Pressure ulcer malignancy in spina bifida patients may thus not present with the classical descriptions, and whereas the current rarity of Marjolin's ulcers in spina bifida patients may be partially explained by the fact that not many spina bifida patients have survived long enough to develop this complication in the past, these peculiar presentations of the Marjolin's ulcers is more difficult to explain. The extent to which the congenital immobility, incontinence and lack of sensation, (factors that predispose to pressure ulcer development in both spinal cord injured patients and those with spina bifida), differs from the same factors when these develop secondary to trauma or tumors, is difficult to determine, but may be another variable that could explain the low incidence of pressure ulcer malignancy in spina bifida patients.

It is conceivable that our environment will see more such survivors, and lack of preparedness for prevention of pressure ulcers may lead to increased numbers with Marjolin's ulcers. Prevention is better that cure, more so when the cure is not possible, especially in an environment such as rural Kenya. All chronic ulcers should undergo multiple biopsies, to help define their therapy, and to avoid missing malignant ulcers [1,18].

## Conclusion

The multifactorial theory best explains the malignant degeneration of pressure ulcers, independent of the cause. Appropriate Marjolin's ulcer patient prognostication should aid in clinical decision making, especially the utilization of resources in poor income countries.

There is need for spina bifida patients and their guardians/caretakers to receive a close follow-up throughout life; health education focused on pressure ulcer prevention as well as early treatment of pressure ulcers when they occur, will avert the development of Marjolin's ulcers, and save lives.

## Competing interests

The author declares he has no competing interests. No grants were given for this work, and no financial benefits are expected from this work. This paper has not been presented in any form, in any forum. There is no association between the author with any commercial firm, and no grants were granted for this article. There are no competing interests in the publication of this article.

## Consent statement

Publication of these cases without patients consent was exempted by the AIC Kijabe hospital ethics committee as the patients consent for publication could not be obtained.
